# Body ownership increases the interference between observed and executed movements

**DOI:** 10.1371/journal.pone.0209899

**Published:** 2019-01-03

**Authors:** Dalila Burin, Konstantina Kilteni, Marco Rabuffetti, Mel Slater, Lorenzo Pia

**Affiliations:** 1 Smart Aging Research Center, Institute of Development, Aging and Cancer, Tohoku University, Sendai, Japan; 2 EVENT LAB- Experimental Virtual Environments for Neuroscience and Technology Laboratory, Department of Clinical Psychology and Psychobiology, Universitat de Barcelona, Barcelona, Spain; 3 Biomedical Technology Department, IRCCS Don Carlo Gnocchi Foundation, Milan, Italy; 4 Institute of Neuroscience, University of Barcelona, Barcelona, Spain; 5 Department of Computer Science, University College London, London, United Kingdom; 6 SAMBA- SpAtial Motor and Bodily Awareness research group- Department of Psychology, University of Turin, Turin, Italy; 7 NIT- Neuroscience Institute of Turin, Orbassano- Turin, Italy; Universita degli Studi di Udine, ITALY

## Abstract

When we successfully achieve willed actions, the feeling that our moving body parts belong to the self (i.e., body ownership) is barely required. However, how and to what extent the awareness of our own body contributes to the neurocognitive processes subserving actions is still debated. Here we capitalized on immersive virtual reality in order to examine whether and how body ownership influences motor performance (and, secondly, if it modulates the feeling of voluntariness). Healthy participants saw a virtual body either from a first or a third person perspective. In both conditions, they had to draw continuously straight vertical lines while seeing the virtual arm doing the same action (i.e., drawing lines) or deviating from them (i.e., drawing ellipses). Results showed that when there was a mismatch between the intended and the seen movements (i.e., participants had to draw lines but the avatar drew ellipses), motor performance was strongly “attracted” towards the seen (rather than the performed) movement when the avatar’s body part was perceived as own (i.e., first person perspective). In support of previous studies, here we provide direct behavioral evidence that the feeling of body ownership modulates the interference of seen movements to the performed movements.

## Introduction

Body ownership is described as sensing our body as the unique source of feelings that we subjectively experience as ours [1]. It is a key component of human self-consciousness and it stands at the root of human nature [2].

Body ownership is thought to rely mainly on afferent information, namely it emerges whenever multisensory incoming signals that constantly reach the body are integrated in both spatial and temporal terms [3–5]. In static conditions, for instance, sensing that “this body is mine” arises from the integration of visual and proprioceptive/tactile inputs but also from other signals such as vestibular and interoceptive input [6,7]. During passive and active movements, sensing that “this moving body is mine” arises also from the integration of further signals such as kinesthetic and muscle contraction information. It is worth emphasizing that such feelings are strictly necessary in order to successfully achieve any kind of willed action. Indeed, in ecological but also in experimental contexts, body ownership allows the estimation of limb positions [8], it provides feedback useful to tune a variety of motor commands [9], it gives key information to adjust errors [10], and so on.

Notwithstanding the above-mentioned considerations, scientific evidence demonstrating whether, how and to which extent body ownership has a role in voluntary action remains scant. In intact brain functioning, it has been reported that embodiment of a real-sized virtual avatar can modulate participants’ experience of agency [11,12]. Within a neuropsychological perspective, earlier studies investigated in general the possible relationship between body ownership and motor system in a variety of clinical populations as, for instance, hemiplegia [13], spinal cord injury [14,15], focal hand dystonia [16] or body identity integrity disorders [17]. In particular, Zampini and colleagues [18] reported that disorders of bodily representations (e.g., personal neglect, anosognosia for hemiplegia, somatoparaphrenia or supernumerary phantom limbs) are often associated to illusory movements of the contralesional arm. Similarly, Preston and Newport [19], described a patient with sometimes experiencing the embodiment of another person’s arm as well as a sense of agency over its movements. Crucially for our purpose, Garbarini and colleagues [20] specifically investigated whether an altered body ownership due to brain damage can influence the sense of agency (i.e., being aware of intending, initiating and controlling our own willed movements [21]) and motor performance. The authors capitalized on a delusion of body ownership in which hemiplegic patients treat, and care for, someone else’s hand as their own [22]. They asked these patients to execute a given action (which cannot actually be achieved because of the severe motor deficits). At the same time, the alien-embodied hand actually performed this action. Results showed that some patients with a delusional sense of body ownership not only embodied the experimenter’s hand but they also misattributed the seen action to their own will, which clearly interfered with the motor performance of their intact hand. This study demonstrated that the pathological embodiment of someone else’ arm entails also the sense of agency over its movements and can interfere with normal motor performance. Concerning the possible role of body ownership on motor performance in healthy participants, some evidence seems to suggest that this might be the case. Typically, these studies display an artificial or virtual limb [23] at a different position from the real limb and they examine the participants’ motor performance while they experience the illusion of owning the fake limb. For example, Newport and colleagues [24] showed that when participants experienced ownership over an artificial limb displayed to the left of the hidden real one, they committed errors in subsequent reaching movements—errors that were consistent with planning the movement from the initial position of the artificial hand, rather than that of the real one. Similarly, Zopf and co-workers [25] found effects on both endpoint errors and initial movement direction when the participants performed pointing movements while experiencing ownership towards a rubber hand. A recent study [26] showed that it is the position of the embodied limb and not that of the real limb that is taken into account when the motor system generates predictions about the sensory feedback of self-generated movements (see [27,28] for similar results). In addition, two recent TMS studies, showed a decrease in corticospinal excitability under a body ownership illusion [29,30] further pointing to a strong link between body and motor representations.

Although all these previous studies with healthy participants suggest that there is a relationship between body ownership and sensorimotor responses, here we were interested in testing how body ownership affects a continuous motor performance and with a more ‘real world’ situation as drawing. By capitalizing on Virtual Reality (VR), we experimentally manipulated both body ownership (i.e., virtual body displayed in a first or third person perspective [31–33] and the congruency between seen and performed movement (i.e., trajectories correspondent or not to the performed movement [10,34]. In other words, the seen movements could be performed by the participants’ virtual body or a virtual body representing another person in the virtual environment and the movements could correspond or not to the performed ones. Following from previous studies, our hypothesis was that we would observe a greater influence of the seen movements to the performed ones when the seen limb was experienced as one’s own hand (i.e., in first person perspective), leading participants to adjust their drawing trajectories towards the seen one. On the contrary, we did not expect to see this pattern with a low degree of body ownership (i.e., in third person perspective).

## Materials and methods

### Participants

Thirty right-handed [35] participants (16 females; mean age: 25 years, range: 19–37 years) participated in the study. All of them provided their written informed consent to participate in this study, which has been approved by the Comité Ético de Investigación of the University of Barcelona, according to institutional ethics and national standards for the protection of human participants. The experiment was a within-subjects design, where each participant experienced both conditions (i.e., first-person perspective or third-person perspective) in a counter-balanced order (participants were randomly assigned to one of the two conditions first). After completing both experimental conditions, participants were compensated with 7 euros, and debriefed about the purposes of the study. It is worth noting that, for ethical reasons, two weeks after the experiment, all participants were contacted by email and asked about their experience in this experiment and whether they had any positive or negative thoughts about it. None of the participants experienced any negative post experimental sensations.

### Equipment

Participants were asked to sit comfortably on a chair with both their own hands placed on the table in front of them. Six reflective markers grouped in two trackable objects were attached on the participants’ right upper limb, in correspondence with the back of the hand and the elbow. The participant’s right hand was placed on top of a Wacom Intuos4 Medium graphics tablet (active area 30 x 46 cm). Then they put on an nVisor SX111 Head-Mounted Display (HMD). Head tracking was performed by a 6-DOF Intersense IS-900 device and arm tracking by the Natural Point’s Tracking Tools system. Finally, all experiment instructions were heard through a Yamaha Digital Sound Projector YSP-4000 powered by a loudspeaker.

The virtual environment was modelled in 3D Studio Max 2012 and implemented in Unity3D (for details of technical equipment see S1 Table): it depicted a virtual reproduction of the real setting, composed by the laboratory, the chair, the table, and the tablet positioned on the right of the participant’s sagittal midline. Depending on the gender and the condition assigned, participants were exposed to one of the scenes at a time, displayed in stereo within the HMD.

### Experimental procedures

As soon as the experiment started, participants were asked to look down towards the table on which their hands were resting (which was registered with the real table) and describe what they saw. In the first person perspective (1PP) condition, they could see a life-sized gender matched virtual body that substituted and was spatially coincident with their real body. In the third person perspective (3PP) condition, participants saw the virtual body opposite and facing them, i.e., 180 degrees rotated, with a partial collocation of the hands in a mirror fashion. Previous studies have shown that a 3PP view of the fake body (or body-part) does not induce the ownership illusion [32,36]. Employing this condition allowed us to manipulate the sense of body ownership only, while maintaining the sense of agency (see below). After this familiarization phase, which lasted 4 minutes approximately, the screen turned black and the participants were given a pen stylus to hold with their right hand, which was connected to the graphics tablet computer.

**Fig 1:**
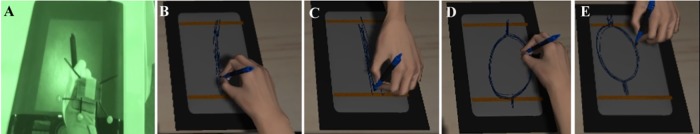
Experimental phases. Participants were asked to draw straight vertical lines continuously and without interruption using a pen on top of a graphics tablet (Fig 1A). During the baseline phase, participants saw the virtual hand drawing the same lines as they drew either in 1PP (Fig 1B) or in 3PP (Fig 1C). During the deviation phase, the virtual hand was seen to draw ellipses and not lines either in 1PP (Fig 1D) or in 3PP (Fig 1E).

Each experimental condition included three different phases during which participants were instructed to fix their gaze on the virtual arm (Fig 1).

During the *baseline* phase participants were asked to draw straight vertical lines with their right hand, continuously and without interruption along the sagittal direction between two orange lines that were marked in the virtual tablet in order to define an active surface where to apply the deviation. At the same time, they were seeing the right virtual arm (in 1PP condition) (Fig 1B) or the left virtual arm (in 3PP condition) (Fig 1C) holding a virtual pen and performing the same drawing at the same time and velocity; in other words, the virtual drawing was the same with the real one, in both 1PP and 3PP. When drawing, the virtual arm was seen to draw on top of a virtual tablet leaving blue traces as it moved (Figs 1B, 1C, 1D and 1E). In 1PP the position of the virtual arm and pen were coincident with the real arm and pen. In 3PP, the position of the pens coincided but the hands were not fully collocated since the virtual arm was rotated by 180 degrees compared to the real one. Each trial was repeated 6 times and lasted 12 seconds.

Then, in the *training* phase, participants were asked to write the alphabet while seeing the virtual arm performing the same movements either in 1PP or 3PP, as before. During this phase that lasted 2 minutes, participants were asked to answer verbally a brief questionnaire with respect to their current experience (see Table 1).

**Table 1:** Questionnaires and results of subjective reports during training phase. Medians and interquartile ranges of the training phase items rated on a 1 to 7 Likert scale, p-values and effect sizes of the within groups comparisons between 1PP and 3PP.

		1PP (n = 30)		3PP (n = 30)			
*During the training phase…*							
	Items	MEDIAN	IQR	MEDIAN	IQR		p value/effect size
**i1**	I feel as if I’m looking at my own hand	6	2	2	3		p<0.000/PS_dep_ = 1
**i2**	I feel as if the virtual arm belongs to another person	2	2	6	3		p<0.000/PS_dep_ = 1
**i3**	The virtual hand moves just as I want, as if it’s obeying me	7	2	5	2		p = 0.002/PS_dep_ = 0.919
**i4**	I feel as if the virtual hand is controlling my will	1.5	1	2	2		p = 0.046/PS_dep_ = 0.589

Finally, during the *deviation* phase and while subjects were again instructed to perform straight vertical lines as before, the virtual arm was seen to draw ellipses instead of lines but at the same rhythm as the participants’ movements (Fig 1D and 1E). This was achieved by applying an online azimuth deviation in the real tracked trajectory data and animating the virtual limb, so that this was seen to draw clockwise ellipses with the center of the tablet as approximate center. Specifically, the depth and azimuth value of the center of the ellipses were defined with respect to the real hand’s position in the first frame of the deviation. In this manner, the ellipses maintained the same relationship with the real hand position, even if the hand was slightly translated when beginning the trial. The ellipses had smaller and larger radiuses, 4 and 7cm, respectively, enough to be consciously detected by the subjects. The depth position of the virtual hand reflected the true depth position of the real hand, while the azimuth position was manipulated. The temporal relationship between the performed and the seen movement was held constant; that is, if participants were moving *x* cm in front and stopped, the virtual limb was seen to move along the arc that corresponded to *x* cm in the depth plane, and stopped. The seen ellipses were always the same except for the fact that slight disturbances in the rigid bodies due to participants’ movements, permitted a more natural view of the ellipses, i.e. not drawing at the exact same trace within each trial. The applied deviation in 1PP and 3PP was exactly the same: given that the position of the pen was congruent in 1PP (where the virtual hand overlapped the real one) and in 3PP (where the hand was 180 degrees rotated) there were no differences between the traced virtual ellipses in both conditions, so we were able to manipulate body ownership only, independently of the visual feedback.

As in the baseline phase, each trial of the deviation phase lasted 12 seconds and the task was repeated 6 times. After the experimental condition was finished, the HMD was removed and participants were asked to complete a questionnaire related to their experience during the deviation phase (see Table 2). Afterwards, they again donned the HMD and the same procedure was repeated for the other experimental condition.

**Table 2:** Questionnaires and results of subjective reports after deviation phase. Medians and interquartile ranges of the deviation’ phases items rated on a 1 to 7 Likert scale, p-values and effect sizes of the within groups comparisons between 1PP and 3PP.

		1PP (n = 30)		3PP (n = 30)		
*After the deviation phase…*						
	Items	MEDIAN	IQR	MEDIAN	IQR	p value/effect size
**i5**	While I was drawing lines, I felt as if the virtual hand was my real hand	5	4	2	4	p = 0.012/PS_dep_ = 0.800
**i6**	While I was drawing lines, I felt as if the virtual arm belonged to another person	3.5	4	5.5	4	p = 0.001/PS_dep_ = 0.951
**i7**	While I was drawing lines, I felt as if the movement of the virtual hand was my movement	3	4	3.5	4	p = 0.493
**i8**	While I was drawing lines, I felt as if the virtual hand was controlled by another person	5	4	4.5	4	p = 0.259
**i9**	While I was drawing lines, I felt as if I was drawing circles/ellipses	6	2	6	3	p = 0.366
**i10**	While I was drawing lines, I felt as if my real right hand disappeared	3	3	3	5	p = 0.400
**i11**	During all the experiment, I felt as if the virtual body I saw was my real body	5	2	2	3	p<0.000/PS_dep_ = 0.999
**i12**	During all the experiment, I felt as if I had more than one body	2	2	2	4	p = 0.016/PS_dep_ = 0.640

When both experimental conditions were finished, all participants were informally asked whether they detected any inconsistency in the movements they saw. All of them verbally reported that the virtual arm was performing ellipses/circles and not lines. We further asked them whether this complicated their task. All participants reported that the task to draw straight vertical lines was very clear to them and that their performance was in line with the request (i.e. drawing lines).

#### Response variables: Subjective ratings

Participants were asked to rate on a 1 to 7 Likert scale items presented in a fixed order concerning their experience, including their feelings of body ownership and agency, both during the training phase, i.e. when the virtual arm moved spatiotemporally congruent with their real arm (Items i1-i4, Table 1) and immediately after the deviation phase, i.e. when this was seen to deviate spatially (Items i5-i12, Table 2).

#### Response variables: Ovalization index

The participants’ movement trajectories during the 6 trials of the baseline and the 6 trials of the deviation phase (12 seconds each trial) were automatically recorded by software built in Matlab connected to the graphics tablet. For each trial, the ovalization index (hereinafter OI), defined as the percentual ratio between the standard deviation of the lateral component of the right-hand trajectory and the standard deviation of the vertical component of the right-hand trajectory, was extracted with the same method previously used [37]: the OI can be qualitatively described as the percentual eccentricity of the drawn trajectory. According to this, the OI is adimensional.

The first action to be performed on the raw measurements was to remove behavioral effects that are likely to arise during blindfold drawing and which can affect the computation of the OI (see also S1 Fig). Particularly, two sequential pre-processing steps are performed on any trial in order to remove slow cycle-to-cycle lateral drawing drift and inclination of the drawing relative to the tablet frame:

1. Removal of any cycle-to-cycle lateral drift (an 8th-order polynomial fitting the time course of the lateral coordinate was first identified and then removed from the coordinate itself) for each participant separately;
2. Reorientation of reference axes in order to identify ‘vertical’ and ‘lateral’ coordinates intrinsic to the recorded movements.

These two actions are valid if a small (less than 45°) rotation compensating for overall drawing inclination has been identified. The resulting drawing trajectory will be therefore characterized by ovalization only (straight lines being a paradoxical null ovalization) and will miss either drifts or rotations.

After removal of confounding factors, the drawing recording is segmented into single cycles (i.e., closed trajectory) starting and ending in the apical vertical points and on the drawn trajectory (x horizontal coordinate, y vertical coordinate) of the i-th cycle the following formula for the computation of an ovalization variable is applied.

$$OI_{i}=\frac{std(x_{i}(t))}{std(y_{i}(t))}$$

Finally, the OI index characterizing the whole performance is obtained as the mean value of the OI calculated about each drawing cycle:

$$OI=\frac{\sum_{i=1}^{N}OI_{i}}{N}$$

The OI index has the following properties:

1. A zero value for straight trajectories (even if misaligned somewhat relative to the tablet’s intrinsic vertical and horizontal axes) without any sign of ovalization;
2. A value of 100 for circular trajectories;
3. A value between 0 and 100 for progressively more oval trajectories.

In case the trajectory of a single cycle is not completed, it is impossible to calculate the OI for that cycle, so these uncompleted cycles were removed from the analysis.

Then, in order to obtain the pure effect of OI in the critical deviation phase, we calculated the ovalization difference (hereinafter OD) defined as the difference of the ovalization indices in the deviation phase from those in the baseline phase, grouped by experimental condition.

### Statistical analysis

Statistical analysis was carried out using Statistica 6.1. Data sets were assessed for normality using the Shapiro–Wilk test. Accordingly, subjective ratings and OD were analyzed with Wilcoxon matched-pair signed-rank test (within-subjects comparisons).

Order effects of conditions in OD were analyzed by means of a 2x2 repeated measures ANOVA with condition (1PP and 3PP) as within-subjects factor and order (i.e. which was the first condition) as between-groups factor. Temporal evolution of the ovalization patterns between trials were analyzed by means of a 2 x 6 repeated measures ANOVA with factors condition (1PP and 3PP) and trial (1, 2, 3, 4, 5 or 6); the same evolution but comparing intervals (corresponding to successive 25% of total number of drawn cycles) were analyzed in a 2 x 4 repeated measures ANOVA with factors condition (1PP and 3PP) and intervals (1, 2, 3, 4). Post-hoc comparisons were conducted with Duncan tests. For each condition, correlations between questionnaire’s items and between subjective ratings and OD were evaluated using a Spearman correlation coefficient. Significance level was always set at p <0.05. Effect sizes for the significant results are given in terms of the probability of superiority of dependent measures, PSdep, defined as the probability that the score from the condition that most frequently has the higher score (Y_1_) will be greater than the score from the condition that most frequently has the lower score (Y_2_), so it refers to an estimator of probability Pr(Y_1_ > Y_2_) [38].

## Results

### Subjective ratings

During the training phase (when the virtual arm was seen to move spatiotemporally congruently with the real arm) in 1PP condition participants experienced the virtual arm as belonging to their body (Table 1, item i1), while in the 3PP condition the reported subjective illusion of ownership score was lower. Analogously, the virtual arm was attributed to another person (i2) in 3PP but not in 1PP. In both conditions, participants experienced agency with respect to the movements of the virtual arm (i3) although higher scores were reported in 1PP. Finally, the control item for agency statement (i4) received low scores in both conditions (Table 1).

With respect to the deviation phase (when the virtual arm performs movements that were spatially incongruent with the real ones), participants in 1PP experienced higher ownership towards the virtual arm (Table 2, item i5) compared to the 3PP condition, and rejected its attribution to the limb of another person (i6). That is, despite the seen deviation, ownership was maintained during the deviation phase in 1PP. In both conditions, participants reported the same median levels of agency (i7-i8) over the seen movements. This confirms that in 3PP they experienced no ownership but the same level of agency of 1PP, although it should be mentioned that the ratings of agency were relatively low since there was a mismatch between the performed and the seen trajectories. Both conditions led to a subjective feeling of drawing circles or ellipses rather than lines (i9). The full body ownership item (i11) was significantly higher in 1PP than in 3PP while the control items (i10, i12) were rated low in both conditions (Table 2).

### Correlations between questionnaire’s items

Considering the answers to the questionnaire items in the experimental condition only (1PP) the results we found support our conclusions about the subjective experience of virtual embodiment. During the training phase (see Table 1), the statement on arm ownership (i1) was negatively correlated with its control statement (i2) (rs (28) = -0.792, p<0.000) and positively correlated with the statement on agency in the same phase (i3) (rs (28) = 0.414, p = 0.006). During the deviation phase (see Table 2) we found a very similar pattern: arm ownership (i5) was negatively correlated with the ownership control statement (i6) (rs (28) = -0.860, p<0.000), positively correlated with agency (i7) (rs (28) = 0.555, p<0.000), negatively with the agency control statement (i8) (rs (28) = -0.481, p<0.000), positively with the perception of drawing ellipses (i9) (rs (28) = 0.321, p = 0.012) and positively with full body ownership (i11) (rs (28) = 0.391, p = 0.002).

In the control condition (3PP) all correlations between items are in line with results previously described and, especially, they confirm our predictions: as example, arm ownership (i5) is negatively correlated with its control item (i6) (rs (28) = -0.825, p<0.000) but, more importantly for our hypothesis, averaged rating of ownership item (i5) in 3PP condition is extremely low (rating 2 out of 7) and definitively lower (rating 5.5 out of 7) than its control item (i6). For a summary of the above-mentioned correlations, see Table 3.

**Table 3:** Summary of the main correlations described in the text. All correlations are evaluated with a Spearman correlation coefficient.

Items	i1	i5
**i2**	-0.792**	
**i3**	0.414**	
**i6**		1PP: -0.860**; 3PP: -0.825**
**i7**		0.555**
**i8**		-0.481**
**i9**		0.321*
**i11**		0.391**

Correlations are marked significant **p< .01, *p< .05.

### Ovalization differences

**Fig 2:**
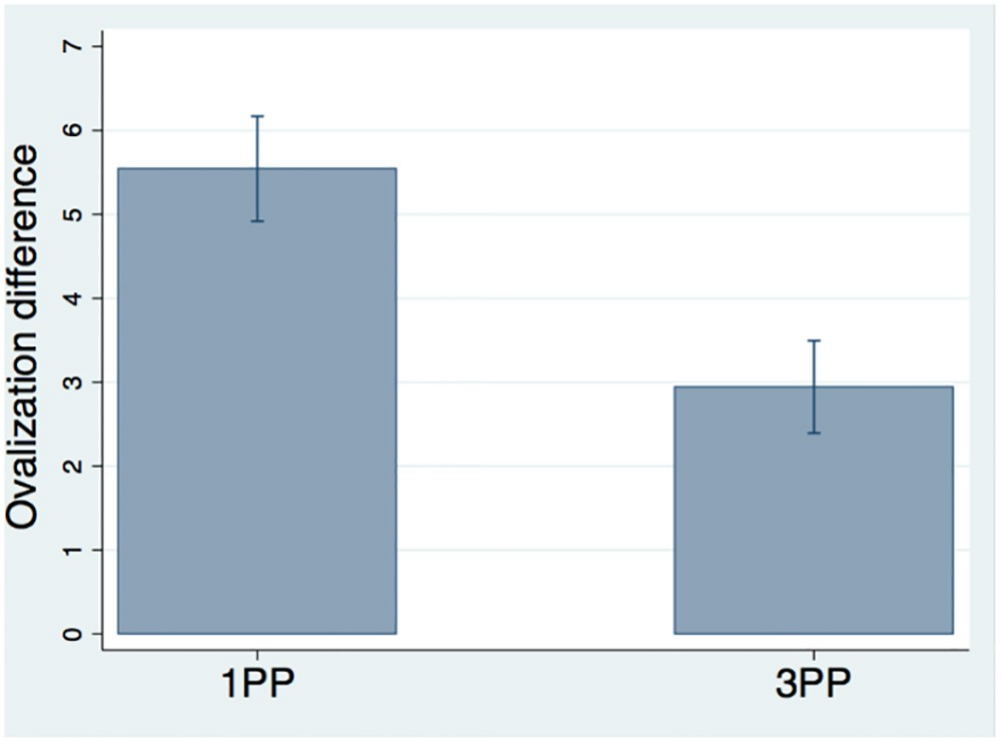
Results of ovalization difference. Means and standard errors for the OD (OI in deviation minus OI in baseline phase) in the two conditions. The OD was significantly higher in the 1PP condition compared to the 3PP (p = 0.001, PSdep = 0.933).

Fig 2 shows the ovalization difference: the 1PP condition led to a significantly higher OD, compared to the 3PP.

As described in the OI method, we did not include in the analysis the cycles without a closed trajectory (11,35% of the total number of the produced cycles by all participants in all conditions and phases).

### Order effect of conditions

There were no order-effects, i.e. no distinction in OD comparing the half of the sample who started with 1PP and those who started with 3PP: analysis revealed a significant effect of condition (F(1,28) = 44.946, p<0.001), but no significant main effect of order (F(1,28) = 2.405, p = 0.132) and no significant interaction between condition and order (F(1,28) = 0.047, p = 0.990). Post-hoc test revealed that the OD was significantly greater in 1PP compared to 3PP (p<0.001), independently from the order of the conditions.

### Temporal evolution of ovalization

We additionally analyzed how the participants’ OD pattern varied during the trials. We extracted the OI for each of the 6 trials per each condition, phase and participant, and we calculated the OD (i.e., deviation minus baseline phase) for each trial separately. There was a significant main effect of condition (F(1, 28) = 32.080, p = 0.001), no effect of trial (F(5, 140) = 0.108, p = 0.990) and no significant interaction of condition by trial (F(5, 140) = 0.994, p = 0.424). Post-hoc comparisons revealed that the OD was significantly greater in 1PP compared to 3PP (p<0.001), independently of the trial.

We also analyzed how the participants’ OD pattern varied along time during the cycles (i.e., as a percentage of the number of cycles drawn, independently from trials). To do so, we first analyzed the number of cycles drawn by participants in each phase for each condition (Table 4), given that each participant may have drawn a different number of cycles (i.e. closed trajectories) per trial.

**Table 4:** Means and standard deviations of the amount of completed cycles drawn for each condition in each phase. At Wilcoxon-matched pair test, no significant differences were found.

	Training phase		Deviation phase	
	1PP	3PP	1PP	3PP
**Mean (SD)**	28.46 (10.36)	26.53 (11.27)	30.66 (11.34)	28.86 (14.91)

Since there were no significant differences in the number of cycles drawn between the two conditions (Wilcoxon matched pairs test, p = 0.245), we calculated the OD for four different intervals that corresponded to the drawing of successive 25% of total number of cycles drawn. There was a significant main effect of condition (F(1, 29) = 38.594, p<0.001), no effect of interval (F(3, 87) = 0.889, p = 0.450) and no significant interaction of condition by interval (F(3, 87) = 1.038, p = 0.380). Post-hoc test revealed that the OD was significantly greater in 1PP compared to 3PP (p<0.001), independently from the interval.

### Correlations between ovalization and subjective ratings

**Fig 3:**
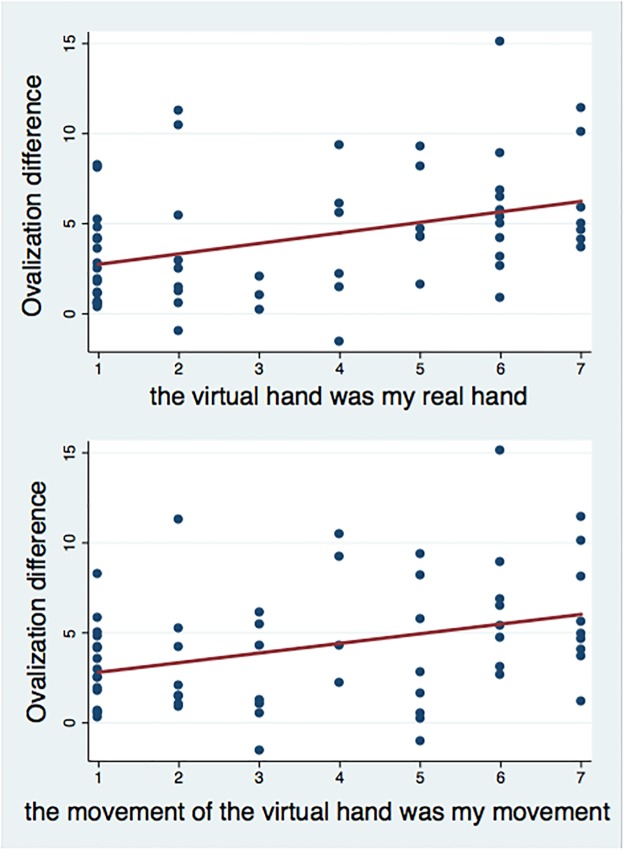
Results of correlations between OD and subjective scores. Scatterplot of OD (deviation minus baseline phase) with arm ownership (i5) and agency (i7) during the deviation phase, respectively.

Considering the results of the questionnaire only in 1PP condition, the OD was positively correlated with scores on arm ownership (i1) (rs (28) = 0.370, p = 0.004) and negatively correlated with scores of arm ownership control during the training phase (i2) (rs (28) = -0.311, p = 0.013). That is, the more they experienced ownership towards the virtual arm before the deviation, the more the participants ovalized during the deviation phase. Moreover, during the deviation phase a positive correlation was found between the OD and feelings of arm ownership (i5) (rs (28) = 0.421, p<0.000), agency (i7) (rs (28) = 0.334, p = 0.009), feelings of drawing ellipses (i9) (rs (28) = 0.323, p = 0.011) and full body ownership (i11) (rs (28) = 0.294, p = 0.022), while a negative relationship was detected for control arm ownership (i6) (rs (28) = -0.469, p<0.000). In other words, the more they experienced arm ownership, full body ownership, agency and illusory sensation of drawing ellipses during the deviation phase, the more the OD (Fig 3).

The results so far indicate that both the OD and the subjective experience of the illusion differ significantly between the two conditions and are correlated between each other, i.e., increasing the feeling of owning the virtual arm and the virtual movement, it increases the ovalization pattern.

In order to investigate whether the effect on OD is due to the condition alone (i.e. a tendency to ovalize more in 1PP than in 3PP, independently of body ownership) or further mediated by the illusion (i.e. a tendency to ovalize more when the seen hand is perceived as one’s own hand, independently of the visual perspective), we computed the partial correlations, partialling out the effect of condition. If condition is the principle determinant of the OD, then the partial correlation between subjective experience and OD should not be significant if the effect of condition is removed.

The partial correlation between the OD and arm ownership during the deviation phase (i5) revealed that ownership scores were significantly correlated with OD (r (28) = 0.421, p<0.000), when controlling for condition. Analogously, partialling out the effect of ownership, the OD was significantly correlated with condition (r (28) = -0.314, p = 0.016).

Similarly, agency (i7) scores were significantly correlated with the OD (r (28) = 0.334, p = 0.009), when controlling for condition, while partialling out the effect of agency, the OD was significantly correlated with condition (r (28) = -0.391, p = 0.002).

In addition, the scores for drawing ellipses (i11) were significantly correlated with the OD (r (28) = 0.294, p = 0.022), when controlling for condition, while partialling out the effect of drawing ellipses, the OD was significantly correlated with condition (r (28) = -0.383, p = 0.003).

## Discussion

In this study, we investigated whether and how body ownership affects motor performance and motor awareness when there is incongruence between the seen and the performed movements. Based on recent neuropsychological evidence that altered body ownership can affect motor representations [18–20], we hypothesized that the experimental manipulation of body ownership in intact brain functioning by means of virtual reality would have modulated the effect of seen movements on performed movements. We found that when healthy participants experienced a virtual hand as their own hand, the seen ellipses drawn by the virtual limb attracted the lines the participants were instructed to draw with their real hand. As a result, participants drew ovalized lines and, importantly, this ovalization was proportional to the experienced body ownership. Therefore, Deviations between the seen and the performed movements led to greater interference effects when the seen hand was perceived as part of participants” own body.

The phenomenon of motor interference has been previously shown between the execution of a movement and the observation of a different movement performed by another person. For instance, if one subject is performing vertical movements while seeing another one performing horizontal movements, the movements of the first will be, in spatial and temporal terms, affected by the second one [39–43]. This interpretation is corroborated by neuroimaging and neurophysiological evidence showing that specific brain areas including the premotor and the parietal cortices are activated by both action execution and action observation [41,43]. Our results do confirm the existence of such kind of interference when observed and executed movements do not match. Most importantly, however, they clearly demonstrate that the interference is significantly stronger when the seen end-effector is perceived as part of the participant’s own body.

Our results built upon findings from previous attempts to investigate incongruences between seen, intended [44] and performed movements. In the classic volition experiment from the 1960s, Nielsen [10] asked healthy participants to draw a straight line following a reference line. In one condition, participants saw the experimenter’s hand instead of their own one, projected onto a mirror so that they believed they were seeing their own hand. Whenever both hands followed the reference line, participants experienced a sense of control over the movements of that hand. However, when the experimenter’s drawing curved rightwards, participants drew leftwards to compensate for the seen deviation, the reported a loss of control over their limb and impressively the majority of them did not question the ownership of the seen hand. Our study replicates these previous findings on motor interference but importantly it shows that this influence is weaker when the seen limb is not perceived as one’s own limb.

Why should body ownership towards the moving hand significantly affect motor performance more than when the hand is not attributed to the bodily self (but the agent is the same)? Our data suggest that the traditional comparator model of motor control [45] can behave differently, depending on whether the errors between the predicted (i.e. the expected hand’s position) and the actual sensory feedback (i.e. where I see the hand to be) are causally attributed to internal (i.e. the sensorimotor system of the agent) or external causes (i.e. another person or the environment) [46]. We therefore propose that these errors are treated differently as a function of body ownership, with errors being more relevant to the sensorimotor update when these concern one’s own body, compared to errors driven by the same agent but applied to the body of another person. The experience of body ownership towards an external object has been proposed to reveal the perception of a common cause and source underlying all bodily-related sensory cues—one’s own body [5,47,48]. Therefore, body ownership can act as a top-down mechanism that attracts prediction errors to internal causes (i.e. the agent’s own body), while, the sense of agency can be mainly modulated by external signals (i.e., outcome values [49] or prior beliefs [50]). This, in turn, means that a full and coherent sense of agency could be linked to an integration of two distinct sources of signals (i.e., efferences and afferences) weighted according to the availability and need of the context [51–54].

Another interesting finding of the study concerns the subjective feeling of voluntariness: our participants, although being aware of the experimental manipulation (given the noticeable spatial disturbances), and even though reporting the subjective sensation as if they were drawing ellipses while they were performing the task, they were not aware of their real performance. In fact, although on the question on the sensation of performing what the virtual body was doing they reported high scores (i9), after the experimental session they informally reported that their real performance was in accordance with the instruction to draw lines. However, as revealed by the ovalization measure, they were actually drawing ellipses. This suggests a conscious detection of the experimental manipulation but an unconscious motor adjustment. The sensation of drawing ellipses instead of lines could not be explained by the visual feedback of the movement and the drawing, since this was the same in 1PP and 3PP. Previous research has shown that healthy people may underestimate their performance deviations as long as their goal is achieved [34], or can adjust their motor planning to compensate for the seen trajectories, even when they are not aware of the manipulation [10,55]. Our data are in strong agreement with the proposed explanation of illusory movements in anosognosia for hemiplegia suggesting that our conscious awareness of voluntary actions relies on intended, rather than performed movements [56,57].

## Conclusions

In this study, we investigated whether and how body ownership towards a seen hand influences motor performance when the seen movements do not correspond to the performed ones. The deviation between the seen and the performed drawings led to greater interference effects when the seen hand was perceived as part of one’s own body. This finding supports previous findings that body ownership is an active component of sensorimotor control in healthy participants.

## Supporting information

S1 Table Details of technical equipment. (DOCX)

S1 Fig (DOCX)
