# Supplementary material for: Body ownership increases the interference between observed and executed movements
Source: PLoS One. 2019 Jan 3;14(1):e0209899. doi: 10.1371/journal.pone.0209899 (PMC6317814; doi:10.1371/journal.pone.0209899)
Supplement: S1 Table — (DOCX) [file pone.0209899.s001.docx]

**S1 Table. The technical equipment used in the experiment**

| Wacom Intuos4 Medium graphics tablet | http://www.cnet.com/products/wacom-intuos4/specs/ |
| --- | --- |
| SX111 Head-Mounted Display | http://nvisinc.com/product.php?id=48  This has dual SXGA displays with 76°H×64°V degrees field of view (FOV) per eye, totaling a wide field-of-view of 111° diagonal, 102° horizontal and 64° vertical, with a resolution of 1280×1024 per eye displayed at 60 Hz. |
| 6-DOF Intersense IS-900 device | http://www.intersense.com/pages/20/14 |
| Natural Point’s Tracking Tools system | http://www.naturalpoint.com/optitrack/products/tracking-tools/ |
| Yamaha Digital Sound Projector YSP-4000 powered loudspeaker | http://usa.yamaha.com/products/audio-visual/hometheater-systems/digital-sound-projector/ysp-4000_black__u/ ?mode=model |
| 3D Studio Max 2012 | http://www.autodesk.com/products/autodesk-3ds-max/overview |
| Unity3D | http://unity3d.com/ |
