# Supplementary material for: Body ownership increases the interference between observed and executed movements
Source: PLoS One. 2019 Jan 3;14(1):e0209899. doi: 10.1371/journal.pone.0209899 (PMC6317814; doi:10.1371/journal.pone.0209899)

**S1 Figure. Effect of drift removal and axes reorientation**

Two potential confounding factors may interfere with the measurement of a blindfold performance of continuous drawing:

1) A slow drift may occur in a continuous drawing expected to repeat itself on the spot

2) The vertical and horizontal reference direction may not correspond to the vertical and horizontal reference axes of the measuring tablet. Those two potential occurrences are not related to the main effect targeted by the experiments: the ovalization induced by coupling. At the same time they may greatly affect the computation of any ovalization score. Therefore, the scoring algorithm includes preliminary steps, before the computation of the Ovalization Index, to remove drift and to compensate for rotation. In the following figures, examples –extracted from the study data set– are presented. For each trial, six plots are organized on two lines (first line for the drawing trajectories, second line for the time profile of drawing XY coordinates) with three plots each (first with raw data, second with data after removal of the drift, third with data after removal of drift and compensation for rotation). The reported examples are : 1) a trial not affected by any distorting factor, 2) a trial affected by drift only, 3) a trial affected by drift and rotation.

Interestingly the Ovalization Index values do not substantially change, as expected, in the unaffected trial, while they show relevant modifications when the trial raw data are affected by any distortion factor. Therefore, the artifact removal algorithm is always applied and the values concerning the raw data are not recorded.

Trial without substatial artefacts (Subject #13 - Trial: "S13_1ppO_16"). OI, due to artefact removal algorithm, changes from 6.9% to 6.3%


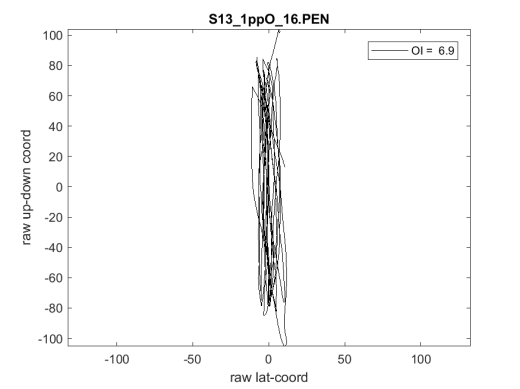

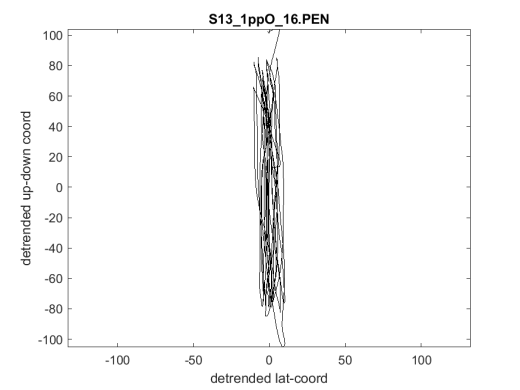

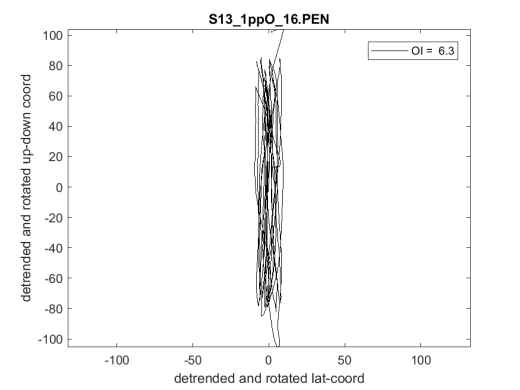


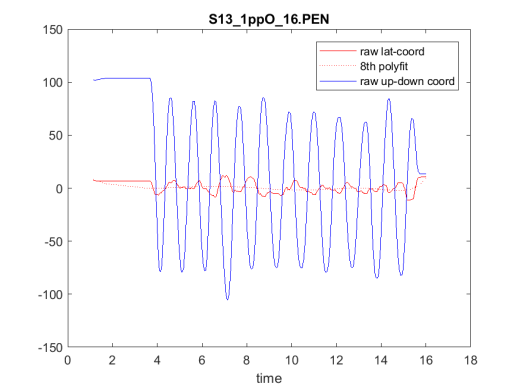

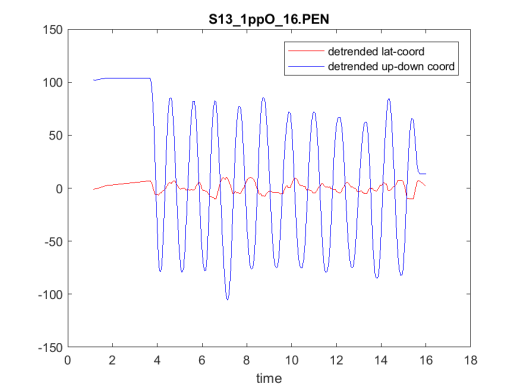

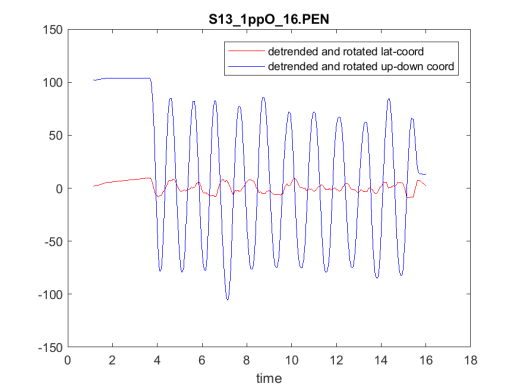


Trial with drift only (Subject #5 - Trial: " S5_1ppI_5"). OI, due to artefact removal algorithm, changes from 5.1% to 3.0%


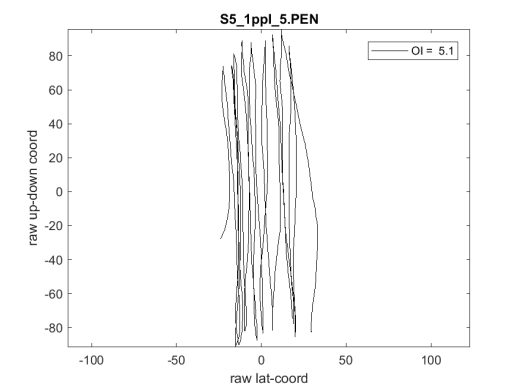

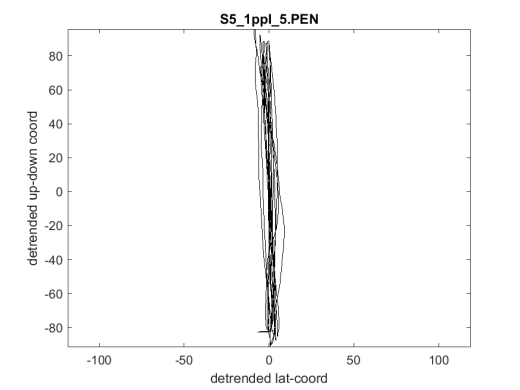

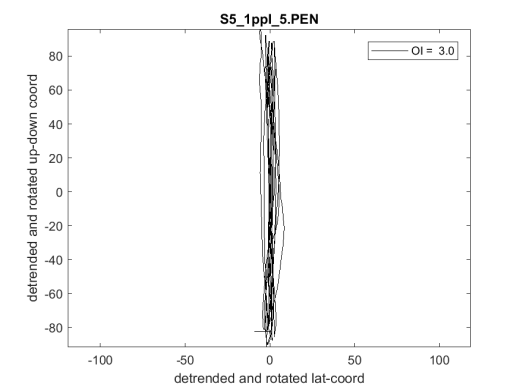


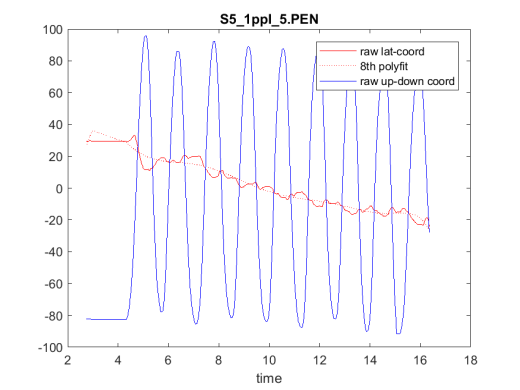

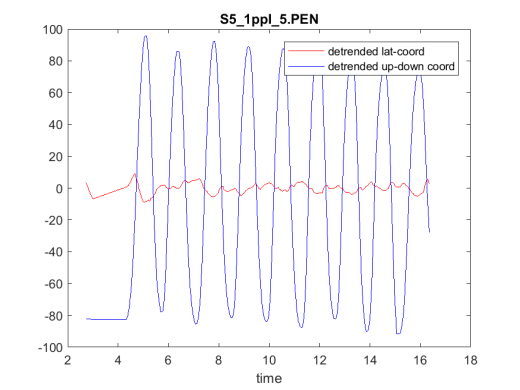

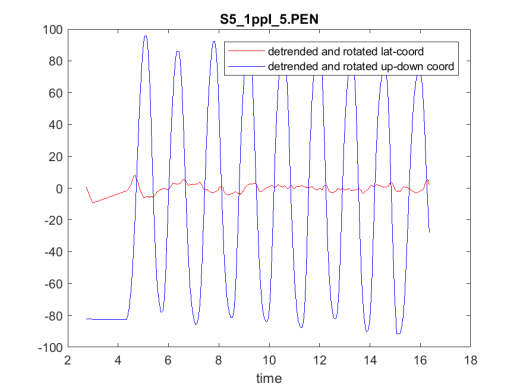


Trial with drift and rotation (Subject #20 - Trial: " S20_1ppO_15"). OI, due to artefact removal algorithm, changes dramatically from 28.7% to 6.2%


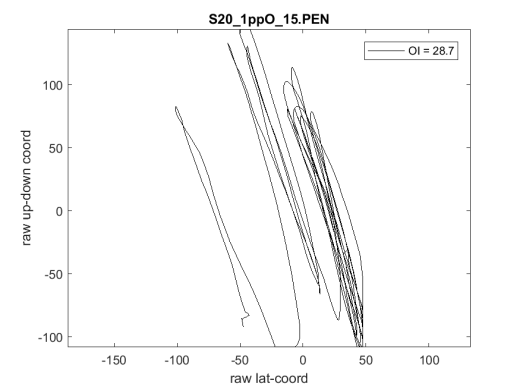

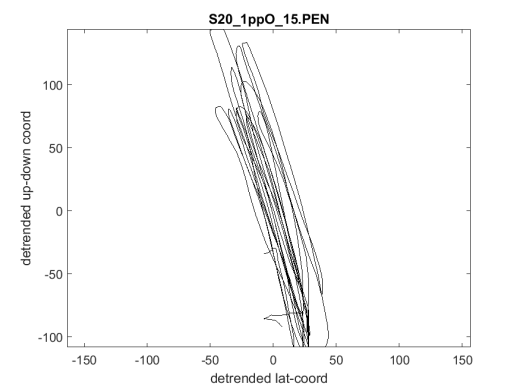

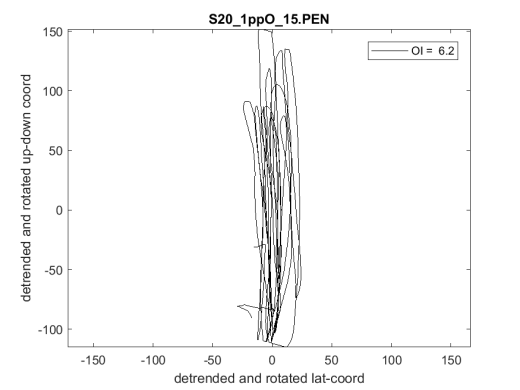


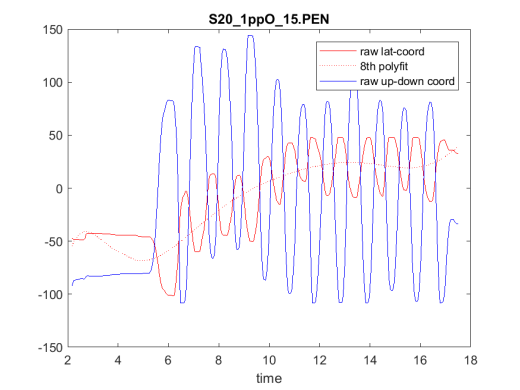

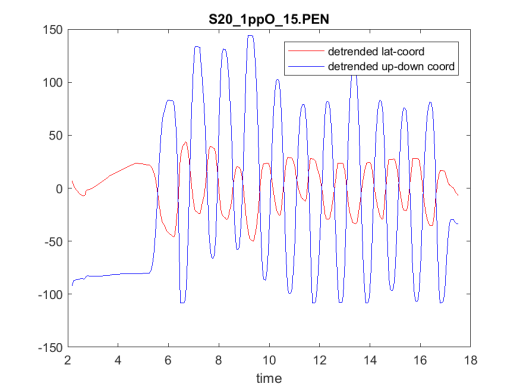

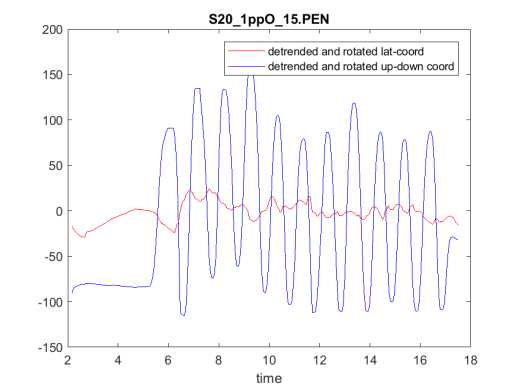

Supplement: S1 Fig — (DOCX) [file pone.0209899.s002.docx]
